# The Acute Chest Syndrome in Cameroonian children living with sickle cell disease

**DOI:** 10.1186/s12887-015-0454-0

**Published:** 2015-09-21

**Authors:** Jobert Richie N. Nansseu, Anastasie Nicole Alima Yanda, David Chelo, Sandra A. Tatah, Hubert D. Mbassi Awa, Judith Seungue, Paul Olivier N. Koki

**Affiliations:** Mother and Child Centre of the Chantal Biya Foundation, Yaoundé, Cameroon; Faculty of Medicine and Biomedical Sciences, University of Yaoundé I, PO Box 1364, Yaoundé, Cameroon

**Keywords:** Acute chest syndrome, Sickle cell disease, Children, Cameroon, Sub-Saharan Africa

## Abstract

**Background:**

Although sub-Saharan Africa (SSA) is particularly affected by sickle cell disease (SCD), there is dearth of research on this topic in the region, specifically targeting the magnitude of SCD-related complications. We therefore conducted this study to determine the burden of acute chest syndrome (ACS) and describe its clinical and therapeutic aspects among SCD children in Cameroon, a SSA country.

**Methods:**

This was a retrospective study carried-out from September 2013 to June 2014 at the SCD unit of the Mother and Child Centre of the Chantal Biya Foundation, a pediatric reference centre in Yaoundé, Cameroon. We enrolled all SCD children with confirmed diagnosis of ACS, and recorded their clinical presentation at admission along with their evolution during hospitalization.

**Results:**

Twenty one cases of ACS were identified during the study period, from 338 hospitalizations of children with SCD. Ages ranged from 11 months to 16 years with a mean (standard deviation) of 5.5 (3.4) years, and a male/female sex ratio of 3.2/1. We noticed relatively low levels of HbF, from 6.4 to 21.9 % with a mean of 14.6 % (6.0 %). The three main symptoms at admission were fever (90.5 %), cough (81 %) and chest pains (28.6 %). Two patients (9.5 %) developed ACS 2 days after admission. The mean values of leukocytes, neutrophils, serum CRP, serum LDH and hemoglobin were respectively 32479.4 (17862.3)/mm^3^, 23476 (11543.7)/mm^3^, 228.2 (132.6) mg/l, 3452.3 (2916.3) IU/l and 6.5 (1.2) g/dl. The main localizations of radiological alveolar consolidations were the lower lobes (90.5 %). Treatment associated broad-spectrum antibiotics (100 %), hydration (100 %), analgesics (43.2 %), whole blood transfusion (66.7 %), and oxygen supplementation (33.3 %). Blood transfusion significantly improved hemoglobin level (*p* = 0.039). The duration of hospitalization, the mean of which was 6.8 (3.1) days, was influenced by none of the tested variables (all p values > 0.05).

**Conclusion:**

ACS is frequent among SCD children in our milieu. Its etiologies seem to be multifactorial. Patients’ parents should be educated to recognize early signs and symptoms of the disease, and consult rapidly. Additionally, clinicians must be trained to diagnose ACS, and manage it promptly and efficiently to avoid its related catastrophic consequences.

## Background

Sickle cell disease (SCD), described for the first time by Herrick in 1910, is an autosomal recessively inherited genetic disorder caused by a single point mutation in the gene encoding the β-globin chain of hemoglobin [1, 2]. It is the most widespread and severe monogenetic disorder in the world [3], especially in sub-Saharan Africa (SSA) where it represents a real public health hazard with 1 to 4 % of babies born affected [4]. SCD is associated with multiple acute and chronic complications such as painful vaso-occlusive events, cerebral vasculopathy, priapism, chronic kidney disease, acute chest syndrome (ACS) and pulmonary hypertension among others [5, 6].

ACS is an acute lung injury syndrome that occurs frequently in patients with SCD. Indeed, it is the second most common cause of hospitalization, and the leading cause of death, contributing to almost 25 % of SCD-related mortality [7, 8]. Moreover, nearly half of deaths due to ACS occur in SCD patients less than 20 years of age [9]. Repeated episodes of ACS negatively impact long-term lung function, resulting thereby in chronic lung diseases [10].

ACS is currently defined as a new pulmonary infiltrate on chest X-ray consistent with alveolar consolidation but not atelectasis, in conjunction with at least one of the following clinical findings: fever (>38.5 °C), reduced oxygen saturation or PaO_2_ (<60 mmHg), tachypnea, intercostal retractions, nasal flaring or accessory muscle use, chest pain, cough, wheeze or rales [11, 12]. In 1979, Charache et al. [13] first suggested using the term “acute chest syndrome” for this complication, acknowledging the difficulties in determining its pathogenesis. Till nowadays, the pathophysiology of ACS remains not fully elucidated, yet multiple risk factors have been identified including: male sex, younger age, higher steady-state of leukocyte counts and hemoglobin level, lower hemoglobin F (HbF) concentrations, previous history of ACS, history of asthma, active smoking or environmental smoke exposure. Furthermore, ACS may be more severe in individuals with HbSS as compared with HbSC disease [6, 7, 14–17].

The etiology of ACS is multifactorial. The three primary studied mechanisms include pneumonia or systemic infection, fat embolism, and direct pulmonary infarction from HbS-containing erythrocytes [6, 11, 18]. The management of this condition is still largely determined by the experience of individual practitioners, and currently there are no conclusive randomized controlled clinical trials to guide therapy [19]. The most common therapy includes nonspecific supportive care strategies aimed at hastening recovery to baseline, including: hospitalization, hydration, analgesics, broad-spectrum antibiotics, bronchodilators, incentive spirometry, supplemental oxygen, and blood transfusions [6, 11, 18, 19].

Although SSA is particularly affected by SCD [4], there is paucity of data on this topic in the region, specifically targeting the magnitude of SCD-related complications. This study was thus undertaken, aiming at determining the burden of ACS and describing its clinical and therapeutic aspects among SCD children in a SSA tertiary pediatric health care facility.

## Methods

### Study setting and participants

This was a retrospective study conducted from September 2013 to June 2014 at the SCD unit of the Mother and Child Centre of the Chantal Biya Foundation. This is a pediatric reference Centre located in Yaoundé, the capital city of Cameroon, a SSA country. This tertiary health care facility receives almost 31,000 consultations with almost 7,000 hospitalizations annually, with patients coming from all over the country. Its SCD unit was opened in December 2008, and is used to dealing with routine out-patient consultations as well as daily follow-up of hospitalized SCD children.

We consecutively and exhaustively included all the patients aged below 18 years and hospitalized in the SCD unit during the study period. These were known SCD children and adolescents with a confirmed diagnosis of ACS (at entry or during hospitalization). The diagnosis of ACS was based on a new radiological alveolar consolidation at the chest X ray, alongside clinical signs and/or symptoms, in keeping with Ballas et al. [12], namely: fever (>38.5 °C), reduced oxygen saturation, tachypnea, intercostal retractions, nasal flaring or accessory muscle use, chest pain, cough, wheeze or rales. Patients who did not perform a chest X ray or whose chest X ray did not show any radiological alveolar consolidation were not included in the study. Data were recorded on socio-demographic characteristics, past medical history and events, main complaints and clinical examination at entry, biological and radiological exams (full blood count, thick blood smear, hemocultures, serum CRP (normal < 6 mg/l), serum LDH (normal range 313–618 IU/l)) as well as the administered treatment and evolution during hospitalization.

### Statistical methods

Statistical analyses used SPSS, version 20.0 (SPSS Inc, Chicago, Illinois, USA). Results are expressed as mean (Standard Deviation) or count (proportion) as appropriate. Qualitative variable comparisons used the *χ*^2^ test or equivalents, and that of quantitative ones, the Student ***t*** test for paired samples or non-parametric equivalents. Odds ratios (OR) with 95 % confidence intervals (CI) were used to appreciate the impact of different variables on the duration of hospital stay, and were calculated by logistic regression analyses. A p value < 0.05 was used to characterize statistically significant results.

### Ethical considerations

All the procedures used in this survey were in accordance with the current revision of the Helsinki Declaration. This study received approval from the authorities of the Mother and Child Centre of the Chantal Biya Foundation, and was delivered an ethical clearance by the Ethical Review Board of the Faculty of Medicine and Biomedical Sciences, University of Yaoundé I, Cameroon. As the study was retrospective, we could not obtain participants’ parents/guardians’ consent. Nonetheless, the need for consent was waived by the just-cited institutional review board.

## Results

We recorded on the whole 21 cases of ACS during the study period, on 338 hospitalizations of children with SCD, hence an in-patient prevalence of 6.2 %. Table 1 displays the study population’s general profile. Ages ranged from 11 months to 16 years, with a mean of 5.5 (3.4) years, and males were more represented than females with a sex ratio of 3.2/1 (Tables 1 and 2). Only seven patients had a hemoglobin electrophoresis dating back to less than one year, HbS ranging from 55 % to 86 % with a mean of 75.1 (11.9) %, and HbF, from 6.4 % to 21.9 % with a mean of 14.6 (6.0) % (Table 2). Only 38.1 % of children were regularly followed-up, i.e. were used to visiting the unit for a check-up at least every 3 months. Besides, not more than 33.3 % of our patients had been adequately vaccinated in accordance with their ages, and only 3 children aged 0–5 years (27.3 %) were currently taking an antibiotic prophylaxis (Table 1).

**Table 1:** General profile of the study population

Characteristic	Number (*N* = 21)	Percentage (%)
Age groups		
0 – 5 years	11	52.4
> 5 years	10	47.6
Sex		
Male	16	76.2
Female	5	23.8
Region of origin		
Adamawa	1	4.8
Centre	11	52.3
Littoral	1	4.8
North-West	2	9.5
South	3	14.3
West	3	14.3
Regularly followed-up		
No	13	61.9
Yes	9	38.1
Adequately vaccinated for the age		
No	14	66.7
Yes	7	33.3
Currently taking folic acid		
No	12	52.1
Yes	9	47.9
Currently taking antibiotic prophylaxis For the 0–5 years old (*N* = 11)		
No	8	72.7
Yes	3	27.3

**Table 2:** Summary of quantitative variables

Variable	Min	Max	Mean	SD	Median	IQR
Age (years) *N* = 21	0.92	16	5.5	3.4	5.0	3.4–7
HbS (%) *N* = 7	55	86	75.1	11.9	77.9	66–86
HbF (%) *N* = 7	6.4	21.9	14.6	6.0	14.8	9.7–19.7
Leukocytes (/mm^3^) *N* = 18	10600	73900	32479.4	17862.3	30295	19050–37875
Neutrophils (/mm^3^) *N* = 18	4800	51600	23476	11543.7	24100	11600–28100
Hemoglobinemia (g/dl) *N* = 18	4.9	8.4	6.48	1.15	6.25	5.4–7.6
Serum LDH (UI/l) *N* = 11	1307	10366	3452.3	2916.3	2093	1623–5301
Serum CRP (mg/l) *N* = 14	4.5	432	228.2	132.6	233.5	89–326
Leukocytes^a^ (/mm^3^) *N* = 7	8300	39100	21557.2	11843.3	18900	11000–35200
Neutrophils^a^ (/mm^3^) *N* = 7	5800	29716	16319.3	10892.7	13400	6475–29100
Hemoglobinemia^a^ (g/dl) *N* = 7	7.5	13.9	9.6	2.3	9.4	7.5–10.6
Duration of oxygen therapy (days) *N* = 7	1	5	3.6	1.6	4	2–5
Duration of hospitalization (days) *N* = 21	3	12	6.8	3.1	6	5–9.5

^a^control values

According to our patients’ parents, duration of evolution of symptoms before consultation ranged from less than 24 h to almost 14 days, with a mean of 4.8 (4.2) days. The presenting complaints are reported in Table 3. The three main symptoms were fever (90.5 %), cough (81 %), and chest pains (28.6 %). The main clinical signs we observed were pulmonary consolidation (100 %), an altered general appearance (61.9 %), clinical signs of anemia (57.1 %), a systemic inflammatory response syndrome (57.1 %), and signs of respiratory distress (33.3 %). Two patients (9.5 %) who were initially admitted for vaso-occlusive crises (VOC) developed ACS after 2 days of hospitalization. Results of biological tests showed very high levels of leukocytes, neutrophils, serum CRP and serum LDH. Indeed, their mean values were 32479.4 (17862.3)/mm^3^, 23476 (11543.7)/mm^3^, 228.2 (132.6) mg/l, and 3452.3 (2916.3) IU/l respectively. Contrariwise, we observed relatively low levels of hemoglobin, with a mean of 6.5 (1.2) g/dl (Table 2). The main localizations of radiological alveolar consolidations were the lower lobes (90.5 %). None of the 10 hemocultures performed was positive, and only one of the 15 thick blood smears performed to seek for malaria parasites was positive to Plasmodium species, specifically Plasmodium falciparum (6.7 %; Table 3).

**Table 3:** Clinical features of ACS

	Number (*N* = 21)	Percentage (%)
Duration of evolution of symptoms before consultation		
≤ 48 h	6	28.6
> 48 h	15	71.4
Symptoms motivating consultation		
Fever	19	90.5
Cough	17	81
Thoracic pains	6	28.6
Generalized pains	6	28.6
Abdominal pains	5	23.8
Dyspnea	5	23.8
Asthenia	2	9.5
Referred for management of severe anemia	3	14.3
Priapism	1	4.8
Vomiting	1	4.8
Clinical signs		
Altered general appearance	13	61.9
Pulmonary consolidation	21	100
Clinical anemia	12	57.1
Respiratory distress	7	33.3
Systemic inflammatory response syndrome	12	57.1
Jaundice	2	9.5
Hemoglobinuria	1	4.8
Result of the chest X ray		
Right upper lobe consolidation	2	9.5
Right middle and lower lobes consolidation	2	9.5
Right lower lobe consolidation	12	57.1
Left lower lobe consolidation	3	14.3
Right and left lower lobes consolidation	2	9.5
Hemocultures		
Not done	11	52.4
Negative	10	47.6
Positive	0	0
Malaria parasitemia (by thick blood smear)		
Not done	6	28.6
Negative	14	66.7
Positive	1	4.8

Patients were placed on two to three antibiotics: a beta-lactam (either ampicillin or ceftriaxone) + a macrolide (oral azithromycin) ± an aminoglycoside (gentamicin) (Table 4). Two days after the fever had dropped, patients were switched to oral cephalosporin (cefixim) or amoxicillin accordingly, for a total duration of 10 to 14 days (both intravenously and orally). Two patients (9.5 %) initially placed on ceftriaxone were still presenting fever on the fourth day of antibiotherapy; they were subsequently switched to vancomycin for seven days with a good improvement. Adjuvant therapy included hyperhydration (100 %), paracetamol (100 %) for fever or pains, tramadol (42.9 %) and ibuprofen (23.8 %). Fourteen patients (66.7 %) were transfused with whole blood, and all of them concurrently received an anti-malarial treatment to prevent transfusion-transmitted malaria. All the seven patients (33.3 %) who presented signs of respiratory distress were placed on oxygen, its duration ranging from 1 to 5 days with a mean of 3.6 (1.6) days (Tables 2, 3 and 4).

**Table 4:** Management and outcome

	Number (*N* = 21)	Percentage (%)
Antibiotherapy		
Beta-lactam		
Ceftriaxone (IV: 50 mg/kg/day)	10	47.6
Ampicillin (IV: 50 mg/kg/8 h)	11	52.4
Gentamicin (IV: 3 mg/kg/day)	16	76.2
Azithromycin (oral: 5-10 mg/kg/day)	21	100
Adjuvant therapy		
Tramadol (IV: 1–2 mg/kg/6 h)	9	42.9
Paracetamol (oral or IV: 15 mg/kg/6 h)	21	100
Ibuprofen (oral: 10 mg/kg/8 h)	5	23.8
Hyperhydration (2.5–3 l/m^2^ BS^a^)	21	100
Anti-malarial treatment		
No	7	33.3
Yes	14	66.7
Oxygen therapy		
No	14	66.7
Yes	7	33.3
Transfusion		
No	7	33.3
Yes	14	66.7
Death		
Yes	1	4.8
No	20	95.2
Duration of hospitalization		
≤ 5 days	8	38.1
> 5 days	13	61.9

^a^BS = Body surface; IV = intravenous

A control full blood count was performed only by seven of our patients 48 h after blood transfusion (Table 3). We did not find any statistical difference between the first and second leukocyte counts (*p* = 0.711), this being the same for neutrophil counts (*p* = 0.811). By contrast, we did notice a significant increase in hemoglobinemia (*p* = 0.039). The duration of hospitalization ranged from 3 to 12 days with a mean of 6.8 (3.1) days. One patient of the female sex died on the fourth day of hospitalization, hence a mortality rate of 4.8 % (Tables 2 and 4). While investigating factors that could have impacted the duration of hospitalization, we found that age, sex, regular follow-up, immunization status, duration of evolution of symptoms prior to consultation, transfusion, oxygen, and the antibiotic used were not implicated in the determination of duration of hospitalization (all p values > 0.05 as depicted by Table 5).

**Table 5:** Factors influencing the duration of hospitalization (equal or below 5 days)

Variable	Odds ratio	95 % Confidence interval	*p* value
Age			
0 – 5 years	0.375	0.061 – 2.305	0.268
> 5 years	1		
Sex			
Male	1		
Female	3.3	0.413 – 26.366	0.262
Regularly followed-up			
No	1.042	0.169 – 6.402	0.664
Yes	1		
Immunization status			
Not good for age	0.300	0.045 – 1.993	0.213
Good for age	1		
Duration of symptoms			
≤ 48 h	5.5	0.710 – 42.600	0.115
> 48 h	1		
Transfusion			
No	2.0	0.291 – 13.738	0.410
Yes	1		
Oxygen therapy			
No	6	0.565 – 63.676	0.113
Yes	1		
Antibiotic used			
Ceftriaxone	1		
Ampicillin	1.944	0.322 – 11.756	0.392

## Discussion

This study showed that ACS accounted for 6.2 % of SCD children hospital admissions with almost 2.1 SCD patients presenting ACS per month. This proportion is comparable to the 1.9 ACS episodes/month among hospitalized SCD children in Bruxelles [15], and higher than the 1.4, 0.3 and 0.2 ACS episodes/month respectively reported in Brazzaville, Antananarivo and French Guiana [20–22]. However, our prevalence of ACS is lower than the 10–20 % rate of hospital admissions reported by Miller and Gladwin in their review [6]. Our prevalence may be an underestimate of the real burden of ACS among our children suffering from SCD for some reasons. First, hemoglobin electrophoresis is not systematically performed in our milieu, and many parents neither know their status nor that of their kids. As we considered only known SCD patients, some unknown SCD patients may have presented this condition and escaped from enrolment. Second, due to parents’ financial constraints, we are used to performing just one chest x-ray during hospitalization. As a result, we could have missed all those whose first chest x-ray was normal but who developed ACS later on, given that radiographic findings in case of ACS may progress over time [9, 11, 23]. If it is true that a positive chest x-ray is the key element to define the disease [12], there have been some claims that a single negative chest x-ray cannot exclude the disease [23–25], taking into account that clinical assessment may appear inadequate to identify ACS [11]. It has been also suggested that every SCD patient presenting with fever must undergo a chest x-ray [11, 24].

Consequently, close clinical monitoring alongside serial radiographic evaluation (without ignoring the risk of radiation) is necessary. In fact, it has been shown that ACS may develop 1 to 3 days after admission for VOC as there may exist a close relationship between ACS and VOC [6, 11, 15, 24]. This is truer for adults than children, as the latter usually present with ACS at admission [26]. In our study for instance, only 2 children (9.5 %) developed ACS 2 days after admission, and pains were concomitantly associated in 52.4 % of cases. Some risk factors involved in the development of ACS during hospital stay have been identified such as the male sex, past medical history of ACS, thoracic pains at entry and use of morphine during hospitalization [15, 27].

Although we did not assess risk factors of developing ACS, we observed a male predominance and relatively low levels of HbF, in line with the literature [6, 16, 17]. We also found that fever (90.5 %), cough (81 %) and thoracic pains (28.6 %) were the main symptoms in keeping with previous reports [9, 21, 28], these symptoms being mainly in favor of pulmonary infections. Despite the fact that we did not have our patients’ baseline leukocyte counts (explained by the fact that many of our patients are not regularly followed-up and are from financially-limited families), we noticed very high leukocyte counts [32479 (17862) mm^3^], especially neutrophils [23476 (11543) mm^3^], associated with elevated serum CRP levels [228.2 (132.6]. These elements are also strongly suggestive that infection may initiate or precipitate the development of ACS among our patients, corroborating previous reports elsewhere [15, 16]. However, though blood culture was not performed in all patients, no germ was identified. This result was also reported by Rucknagel et al. [25] who incriminated rib infarction as the main etiology of ACS. But without invasive procedures (bronchoscopy with broncho-alveolar lavage), it is very difficult to rule out conclusively a bacterial infection as a cause of ACS. Microorganisms that have been identified associated with ACS include Streptococcus pneumonia, Chlamydiae pneumonia, Mycoplasma pneumonia, influenza virus A H1N1, parainfluenza virus, respiratory syncytial virus and coronavirus among others [15, 21, 23].

Besides, we observed that radiological abnormalities of the lower lobes (90.5 %) were the prevailing ones, mirroring previous findings [23, 28]. Contrariwise, other studies showed that the main radiological localizations of ACS were the upper lobes in children, and the lower ones in adults [9, 20]. While the upper lobes predominance has been associated with an infectious etiology especially in children, the lower or multi-lobes predominance has been linked to pulmonary thrombosis and fat embolism [9, 16, 20] or rib infarction [25]. Our results are therefore suggestive that the pathogenesis of ACS in our setting is also multifactorial. Further well-designed studies with large sample sizes are warranted to better elucidate the etiology of ACS in our setting.

In line with the literature [6, 19, 22], our management of ACS included broad-spectrum antibiotherapy, hydration, analgesics, supplemental oxygen and transfusion, but none of these supportive care impacted the duration of hospital stay. Actually, only patients presenting respiratory distress were placed on oxygen, mainly due to limitation of resources. Likewise, we transfused only SCD patients who had a hemoglobin level ≤ 7 g/dl given that blood safety remains an issue of major concern in the milieu [29]. Although transfusion did not reduce the hospital stay, it significantly increased the hemoglobin level (*p* = 0.039), perhaps improving therefore the clinical state of our patients. Early transfusion of SCD patients presenting with ACS should be encouraged in SSA settings, especially transfusion of packed red blood cells instead of whole blood to reduce transfusion-related adverse reactions, despite recurrent blood shortages that occur in the region [30]. Nonetheless, more studies are needed to underpin this suggestion with robust scientific evidence and indicate which threshold should be considered to transfuse SCD children suffering from ACS.

The mean duration of hospitalization we found (6.8 days) is comparable to the 7 days-duration reported by Bertholdt et al. [15], but a bit higher than findings from Vichinsky et al. (5.4 days) [9] and lower than what has been reported by Vichinsky et al. in another study [23] and Hunald et al. [21]: more than 10 days and 12 days respectively. Introduction of other supportive care in our practice like bronchodilators and incentive spirometry as elsewhere [6, 19], along with systematic oxygen supplementation and early blood transfusion could substantially reduce the duration of hospital stay. Furthermore, the use of dexamethasone deserves some close considerations. In fact, there is body of evidence bolstering that low dose dexamethasone (0.3 mg/kg/12 × 4 doses) significantly diminished the duration of hospital stay, the need for blood transfusion, oxygen and analgesics use, and duration of fever, without a rebound effect [31] which was observed with high dose methylprednisolone in cases of VOC [32].

One of our patients died, hence a mortality rate of 4.8 % which concurs the 4 % percentage obtained by Bertholdt et al. in Belgium [15]. This was an inadequately-vaccinated and unregularly-followed female SCD patient aged 18 months, with a HbF level of 18.9 %. She was brought to consult for fever, cough and respiratory distress, and the chest x-ray revealed medium and lower right lobes alveolar consolidations. Titers of LDH and CRP were respectively 10366 IU/l and 414 mg/l. She was placed on a triple antibiotherapy (ceftriaxone, gentamycin and azithromycin), oxygen supplementation, and was transfused as well. But she died at day 4 of hospitalization in a context of severe sepsis.

Unfortunately, the retrospective design and small sample size constitute some weaknesses of the present study. Besides, generalization of our results may be impeded by the use of data collected from only one hospital centre. Another limitation is that biological data were incomplete due to parents’ financial limitations, as the study was not sponsored. Nonetheless, in this particularly difficult context, we carried-out serious and reliable data collection and analyses, and have shown that despite the difficulties in managing our patients, the outcome is relatively similar when compared with resource-rich settings in terms of duration of hospital stay and mortality rate. Eventually, and to the best of our knowledge, this is the first study conducted in our milieu targeting ACS burden and presentations.

## Conclusion

ACS is frequent among SCD children in our milieu. Its etiologies seem to be multifactorial including infection, pulmonary thrombosis and fat embolism. After an initial normal chest x-ray, especially in a patient presenting with VOC, repeated clinical evaluation must be conducted and possible changes in the clinical status should indicate the necessity of a new radiographic examination. Patients’ parents should be educated to recognize early signs and symptoms of the disease, and consult rapidly. Additionally, clinicians must be trained to correctly diagnose ACS, and manage it promptly and efficiently to avoid its related catastrophic consequences. Additive treatment such as incentive spirometry, bronchodilators and even low dose dexamethasone could perhaps be introduced in our settings to reduce the hospital stay and hasten recovery. On recovery, treatment with hydroxyurea should be discussed to reduce the likelihood of recurrent episodes.

## Abbreviations

ACS: Acute chest syndrome
Hb: Hemoglobin
SCD: Sickle cell disease
SSA: Sub-Saharan Africa
VOC: Vaso-occlusive crisis
